# Obesity, Visceral Fat, and NAFLD: Querying the Role of Adipokines in the Progression of Nonalcoholic Fatty Liver Disease

**DOI:** 10.5402/2011/592404

**Published:** 2011-08-28

**Authors:** M. S. Mirza

**Affiliations:** SpR Surgery, Ninewells Hospital, 65 Lister Court, Dundee DD2 1UY, UK

## Abstract

Nonalcoholic fatty liver disease (NAFLD) represents a spectrum of clinicopathologic conditions ranging from steatosis alone to nonalcoholic steatohepatitis (NASH), with varying risks for progression to cirrhosis and hepatocellular carcinoma. There is mounting evidence that NAFLD not only complicates obesity, but also perpetuates its metabolic consequences. Critical event that leads to progressive liver injury in NAFLD is unknown. Obesity reflects a generalized proinflammatory state with its increased inflammatory markers like C reactive protein, IL-6, IL-8, IL-10, PAI-1, TNF-*α*, and hepatocyte growth factor. The elevated production of these adipokines is increasingly considered to be important in the development of diseases linked to obesity and the metabolic syndrome. Disordered cytokine production is likely to play a role in the pathogenesis of NAFLD. There is no effective treatment for NAFLD, though weight loss may halt disease progression and revert histological changes, the underlying mechanism remaining elusive. All stages of the disease pathway from prevention, early identification/diagnosis, and treatment require an understanding of the pathogenesis of liver injury in NAFLD.

## 1. Introduction

With the rapidly growing prevalence of obesity [1] throughout the world, morbidity and mortality related to its complications is on the rise [2]. Obesity is considered a gateway disease. Defined and classified by body mass index (Table 1), individuals with severe obesity have a disproportionately high risk of comorbidities including nonalcoholic fatty liver disease (NAFLD), cardiovascular disease, and diabetes [3–6]. NAFLD now represents the most common of all liver disorders and the most frequent cause of chronic liver disease [7]. It is a syndrome with multifactorial aetiology with which obesity is most commonly associated [8]. Obesity itself is typically a heterogeneous condition due to the regional distribution of fat tissue. There is growing evidence that the distribution of adipose tissue in the body is of importance for the development of the metabolic complications of obesity. Adipose tissue as an endocrine organ has become accepted [9, 10] with the distinctive biological properties of visceral adipose tissue presumably contributing to the increased pathogenecity of obesity.

## 2. Obesity and the Metabolic Syndrome

Metabolic syndrome or syndrome X (Tables 2(a), 2(b) and 3), is a constellation of closely related cardiovascular risk factors characterized by obesity, insulin resistance, hyperinsulinemia, hyperglycaemia, dyslipidemia, and hypertension [11–15]. Its pathological basis still remains elusive. Often associated with insensitivity to insulin, obesity is considered a key factor in the development of the metabolic syndrome. In obese individuals, fatty tissue becomes insensitive to the action of insulin resulting in greater breakdown of triglycerides [16]. Overabundance of circulating free fatty acids from excessive adiposity contributes to the development of insulin resistance [16]. Upon reaching insulin sensitive tissues, excessive fatty acids create insulin resistance by the added substrate availability and by modifying downstream signaling [16]. The release of adipose tissue-derived proteohormones called adipokines also comes under the influence of insulin. Under normal weight conditions, adipokines guarantee homeostasis of glucose and lipid metabolism. Their dysregulated production in the obese state is associated with insulin resistance and appears to play an important role in the development of the metabolic syndrome [16, 17]. Resistance to Leptin, an important adipokine has been suggested as an alternative concept to explain the metabolic syndrome [16, 18]. In general, conditions like obesity in which leptin deficiency or resistance are present are associated with triglyceride accumulation in nonadipose organs like liver, muscle, and pancreas [16, 18]. The resulting lipotoxicity in these organs results in diabetes by causing insulin resistance. Leptin also seems to lower insulin secretion [16, 19]. Leptin resistance, however, could relate to the hyperinsulinemia of the metabolic syndrome [16, 20]. 

NAFLD represents the hepatic component of the metabolic syndrome [14, 41]. Insulin resistance is a universal phenomenon in NAFLD [14, 42, 43]. The presence of metabolic syndrome carries a high risk of necroinflammation and fibrosis among NAFLD subjects and remains strongly correlated with disease severity and progression [13, 14]. Specific adipokines may link the metabolic syndrome, type 2 diabetes and NAFLD and an imbalance in adipokine expression could play a pivotal role in disease progression to NASH and cirrhosis.

## 3. Adipose Tissue, Adipokines, and Inflammation

Fat is not uniformly distributed in the body (Table 4). Visceral fat depots are located in the body cavity beneath the abdominal muscles and composed of greater and lesser omentum and the mesenteric fat [23, 25, 44]. A lesser amount of visceral fat is located retroperitoneally [23, 25, 44]. In general, visceral fat accounts for up to 20% of total fat in men and 5–8% in women [44]. The abdominal subcutaneous fat is located immediately beneath the skin and on top of the abdominal musculature [25, 44]. The predominance of lower body fat is subcutaneous most of which is stored in the femoral and gluteal regions [23, 44] (Table 2).

The distribution of fat appears more important than the total fat mass in obesity [23, 45] (Table 2). A predominantly upper body fat distribution increases the risks for the metabolic complications of obesity including hepatic steatosis especially when it is associated with increased intra-abdominal fat [46–49]. Most “metabolically obese” normal weight subjects have some increase in adipose tissue mass and insulin resistance probably due to an increase in visceral fat [25]. Thus, subjects with a relatively low BMI can have gross increases in abdominal visceral fat [25, 50, 51], and others with a high BMI may have very little intra abdominal/visceral fat [25, 52]. 

Adipose tissue comprises of mature adipocytes, preadipocytes, stromovascular cells, connective tissue matrix, endothelial cells, sympathetic nerve fibres, and macrophages which may all contribute to adipose tissue function [26, 49, 53] In addition, it expresses numerous receptors that allow it to respond to afferent signals from traditional hormone systems as well as the central nervous system [49]. The cellular composition of fat can vary substantially according to anatomical location and body weight. The anatomic location of each adipose tissue depot itself affects endocrine function [25, 26, 49]. Fat mass can increase in one of two ways: individual adipocytes can increase in volume, or they can increase in number as more are derived from preadipocytes [44]. Fat cell size is an important determinant of the metabolic activity of the fat depot [45]. Visceral adipocytes are somewhat smaller than subcutaneous cells [54–56], though omental fat cell size does not differ significantly from subcutaneous adipocyte [56]. Enlarged fat cells appear to secrete increased amounts of adipokines [55, 57, 58]. 

The stromal vascular fraction of adipose tissue contributes to the major differences between subcutaneous and visceral fat including adipokine production. The number of stromal vascular cells per gram of adipose tissue are reported to be higher in omental compared to subcutaneous fat possibly to be due to higher number of endothelial cells in the omental fat [59]. On the other hand stromal cells from subcutaneous fat proliferate faster than those from the omental region [59]. Another component of adipose tissue, the preadipocytes, has been shown by some to show greater differentiation capacity in case of subcutaneous fat compared to visceral adipose tissue but not by others [44, 59, 60]. 

The increased fat mass assumes greater significance with recent recognition of the adipocyte as an endocrine organ capable of secreting a variety of bioactive peptides that exert multiple effects at both the local and systemic level [7, 49]. To date, over fifty “adipokines” have been reported to be secreted by adipose tissue that not only influence body weight homeostasis but also inflammation, coagulation, fibrinolysis, insulin resistance, diabetes, atherosclerosis, and some forms of cancer [53, 61]. These include leptin, adiponectin, resistin, acylation stimulating protein, TNF-*α*, TGF-*β*, plasminogen activator inhibitor, angiotensin II, and interleukins 6, 8, 10 [49, 53] to name a few. 

The vascular anatomy and metabolic activity of fat from various depots differ in a way that may explain the association of visceral but not subcutaneous fat with obesity-related cardiovascular and metabolic problems [23, 25, 46, 58, 62] (Tables 5 and 6). Regional differences are pronounced between omental and subcutaneous fat depots [26–29, 53]. The venous drainage of visceral fat is via the portal system, directly providing free fatty acids as a substrate for hepatic lipoprotein metabolism and glucose production [23, 30, 45, 48]. Visceral omental fat has a higher rate of lipid turn over than subcutaneous fat [30, 63, 64] and omental adipocytes have higher basal and adrenaline-stimulated levels of intracellular cAMP [30, 65] being more responsive to the lipolytic effects of catecholamines [31, 64, 66], and less responsive to the antilipolytic effects of insulin [30, 64, 67, 68]. Omental adipocytes express higher levels of glucocorticoid receptors [25, 30, 69], and in very obese individuals express lower levels of lipoprotein lipase protein and mRNA than do subcutaneous adipocytes [30, 70]. Expression of IL-6, IL-8, resistin, PAI-1, MCP-1, and Visfatin is relatively greater in visceral fat compared to subcutaneous fat, whereas leptin, adiponectin, and adipsin are greater in subcutaneous adipose tissue [26, 30–36, 49, 57, 71]. There is no important regional variation of TNF-*α* production [25, 31, 71].

Taken as a whole, these observations suggest that visceral adipocytes may represent a specialised adipocyte population designed to release nutrients rapidly in conditions of stress.

Obesity has been characterized by a state of chronic low- grade inflammation [72, 73]. The basis of this view is an increased circulating level of several inflammatory markers in the obese including CRP, TNF-*α*, IL-6, IL-8, IL-18, MIF, haptoglobin, SAA, and PAI-1 [72, 74–76]. The inflammatory state may be causal in the development of insulin resistance and the metabolic syndrome [72, 76]. It remains unclear as to the extent to which adipose tissue contributes quantitatively to the elevated circulating levels of these factors and whether there is a generalised or local state of inflammation [72]. The increased production of adipokines and acute-phase proteins in obesity is considered to be primarily related to local events within the expanding fat depots [72]. With increasing evidence of the infiltration of adipose tissue by macrophages the nonadipocyte fraction may be a significant component of the inflammatory state within the fat tissue [77, 78]. Why the secretion of adipokines and other inflammation-related proteins from adipose tissue rises sharply with increasing adiposity remains obscure. It has been proposed that relative hypoxia of clusters of adipocytes within an expanding adipose tissue mass triggers the inflammatory response [72].

## 4. Nonalcoholic Fatty Liver Disease (NAFLD)

Nonalcoholic fatty liver disease is a form of chronic liver disease histologically indistinguishable from alcoholic hepatitis occurring in individuals without significant alcohol consumption [79, 80]. NAFLD comprises a morphologic spectrum of liver lesions (Table 1) ranging from steatosis alone to nonalcoholic steatohepatitis (NASH), with varying risks for progression to cirrhosis [38, 79, 81]. Whereas nonalcoholic steatosis without necroinflammatory change is generally a benign condition, NASH defines the turning point in the progression of NAFLD from steatosis to advanced fibrosis and cirrhosis [8]. NASH is characterized by hepatocellular steatosis, necroinflammation, hepatocellular injury, and pericellular or perisinusoidal fibrosis [7, 82]. Isolated portal inflammation/fibrosis (IPF) signifies a subset of individuals with NAFLD who have portal fibrosis associated with hepatic steatosis in the absence of zone 3 hepatocellular injuries [83–89]. It is thought to mark the onset of disease progression [90].

## 5. Histological Criteria for Diagnosis

The principal histologic feature of NAFLD is the presence of macrovesicular fatty change in hepatocytes with displacement of the nucleus to the edge of the cell wall [38, 39, 91]. Additional features present variably are Mallory bodies, ballooning degeneration, predominantly lobular neutrophilic inflammation, and zone III perisinusoidal fibrosis [38, 39, 91]. In many cases, atypical features like predominantly lymphocytic inflammation and/or portal fibrosis may be seen [38, 39, 91].

## 6. Historical Perspectives and Prevalence

In the 1970s, fatty liver hepatitis was noticed to affect morbidly obese patients who had undergone jejunoileal bypass [92]. This was recognised as a new disease entity by Ludwig who in 1980 coined the term nonalcoholicsteatohepatitis (NASH) for it. [93]. Later histopathological changes consistent with the different grades of NAFLD were reported with certain drugs including steroids, amiodarone, and isoniazid [94–96] (Table 7). Increasingly, this entity has been expanded by its recognition as an important differential in the diagnosis of abnormal liver enzymes [97]. The prevalence of NAFLD/NASH appears to parallel the degree of obesity [7, 79, 80, 82, 98].The prevalence of simple steatosis in obese patients is 70–100%, whereas NASH is found in 20–25% and 2-3% have cirrhosis [88, 89, 99–101]. The prevalence of IPF is approximately 29% [12, 14, 24, 27–29, 62]. Many patients with cryptogenic cirrhosis have metabolic risk factors for NAFLD and are likely to represent cases of previously unrecognised NAFLD [102, 103]. In cirrhosis, both steatosis and inflammatory changes may have disappeared making the cause difficult to establish [11, 38, 39].

## 7. Pathophysiology

The liver plays a central role in lipid metabolism, importing serum-free fatty acids (FFA) and manufacturing, storing, and exporting lipids and lipoproteins. However, the patho-physiology that leads to NAFLD is not well understood; in particular, the factors that lead to progressive hepatocellular damage after triglyceride accumulation are not well elucidated. The two metabolic abnormalities most strongly associated with NAFLD are insulin resistance and an increased supply of fatty acids to the liver [104].

The “two- hit” hypothesis remains the leading theory of the pathogenesis of NAFLD [105, 106]. The “first hit,” insulin resistance, leads to steatosis as a consequence to the alterations in lipid metabolism [105, 106]. Insulin resistance results in increased FFA release from the fat stores. Hepatic FFA activity is largely uncontrolled and therefore directly proportional to plasma FFA concentrations. In the liver, FFA can either be oxidised to generate ATP or esterified to produce triglycerides which can be stored or incorporated into very low density lipoproteins for export. Defects in either of these two pathways could lead to hepatic steatosis. Hepatic lipid accumulation does not universally result in liver injury indicating that additional secondary insults are important [105]. Progression to inflammation and fibrosis appears from oxidative stress, the “second hit,” triggered by the accumulation of fatty acids producing more oxidant substances than the antioxidant processes of the liver can handle [105, 106]. Lipid peroxidation and liver damage may be influenced by a variety of factors such as cytochrome P450 2E1 induction, endotoxin, hepatic iron, Kupffer cell dysfunction, and mitochondria changes and ATP homeostasis [38, 105]. Insulin resistance can contribute to all these pathways. Portal inflammation appears to mark the occurrence of this “second hit” [90]. Alterations in the adipokines resistin, leptin, adiponectin, and TNF-*α* are thought also to play a role in the pathogenesis [11, 38] of NAFLD.

## 8. Diagnosis and Assessment of Severity

Most patients with NAFLD are asymptomatic, and typically patients are found incidentally to have abnormal biochemical liver function tests or hepatomegaly when evaluated for another condition [107, 108]. A percentage of patients present with ill-defined symptoms of right upper quadrant pain, abdominal discomfort, fatigue, or malaise [40, 97, 109, 110]. Laboratory tests are nonspecific. Most patients have a moderate elevation in transaminases, and the ALT/AST ratio is usually less than one [108]. A variable elevation of alkaline phosphatase and gammaglutamyl transpeptidase is frequent [108]. Unexplained persistent elevation of ALT is most commonly due to NAFLD once hepatitis C and other chronic liver diseases have been excluded, [91, 111]. Up to 50% have diabetes or glucose intolerance and up to 80% may reveal fasting hypertriglyceridemia [93, 109, 112, 113]. An increase of iron in the liver accompanied by an elevated saturation of transferrin and serum ferritin may be present [109]. Antinuclear antibodies have been noted in 10%–25% of NAFLD patients, the significance of which remains unclear [93, 114, 115].

Imaging studies including ultrasonography, CT scanning, and MRI are useful in demonstrating hepatic steatosis, at least when fat accumulation is moderate to severe [116–119]. However, these tests may underestimate less severe steatosis and cannot detect the differences between NASH and nonprogressive NAFLD [118–122] Liver biopsy remains the gold standard in the diagnosis and staging of NAFLD [7, 82]. It is the only diagnostic test that reliably identifies and quantifies hepatic steatosis, inflammation, necrosis, and fibrosis; and thereby estimates prognosis and disease progression [8, 120, 123–125]. It is recommended during bariatric surgery to assess the extent of liver damage, the behaviour of liver architecture after weight loss, and the relevance of NASH in the evolution to cirrhosis [126, 127]. In addition to confirming the clinical diagnosis, liver biopsy is valuable for excluding other liver disease and for monitoring treatment efficacy [82, 128, 129]. Disadvantages to biopsy include observer variability, sampling variation, and morbidity and mortality [130].

To date, few investigations have specifically addressed sampling error and variability in NASH. Sampling variability has the potential to significantly alter disease grade and stage. The histological findings of NAFLD are usually presumed to be homogenously distributed throughout the liver. Consequently, in clinical practice, a single random core biopsy is usually considered representative of overall hepatic involvement. However, there are no data to support these assumptions in NASH [131].

Broadly, two different types of liver biopsy techniques are in use: core (needle) biopsy and wedge biopsy. Needle biopsy can be obtained percutaneously either blindly or under radiological or ultrasound guidance and by transjugular catheter [130, 132, 133]. Both types of biopsies can be taken during laparoscopic or open surgery [130, 132–134]. Core biopsy has declined considerably at laparotomy or laparoscopy and is now used mainly when focal lesions are discovered during routine surgery [133]. Wedge biopsy of the liver yields more accurate diagnosis of liver diseases than does needle biopsy, but is more invasive and haemostasis more problematic [135].

Types of biopsy needles include: the Tru-Cut, which is a cutting needle, and the Menghini needle, which uses a suction technique [132, 133, 136]. The cutting needle usually produces a larger sample, but is associated with a higher risk of complications. A suction needle tends to produce more fragmented samples [132, 137]. The size of the specimen varies depending on the size of needle: a biopsy obtained with 14-gauge(G) to 21G needle is usually defined as large, whereas needles less than 21G result in thin or fine biopsy samples with a core less than 1 mm in diameter. The size of the biopsy specimen that varies between 1 and 3 cm in length and between 1.2 and 2 mm in diameter represents 1/50,000 to 1/100,000th of the total mass of the liver [130, 132, 133]. The traditional assumption that a sample 1.5 cm long or containing four to six portal tracts, or both, is adequate is no longer true for the grading and staging of chronic liver disease. Studies show that smaller samples significantly underestimate the disease severity and samples at least 2 cm long can ensure greater diagnostic accuracy [133, 138].

In NAFLD, the distinction between steatosis and steatohepatitis and the assessment of the severity rely entirely on liver histology. Small unrepresentative samples in the context of uneven distribution of lesions can result in substantial misdiagnosis and staging inaccuracies. In addition, important pathology can be overlooked if only a single biopsy specimen is taken. It is suggested that three consecutive specimens may improve the diagnostic yield of liver biopsy [139]. While sampling error has been studied fairly extensively in cases of needle biopsy, this is not true in cases of wedge liver biopsy especially in the context of NASH.

## 9. Progression of Liver Disease

Knowledge about the natural history of NAFLD is still evolving. There are several distinct histologic states that indicate progression of the lesion: fatty liver alone, steatohepatitis, steatohepatitis with fibrosis, and eventually cirrhosis [91, 97]. The precise risk of mortality in patients with NAFLD is not known. Preliminary studies suggest a more benign course for simple steatosis and the mortality remains low, although some have shown occasional progression to cirrhosis [38, 40, 140]. Cross-sectional series have shown that 30–40% of patients with NASH have advanced liver fibrosis at the time of presentation [91, 97, 141], whereas 10–15% may have established cirrhosis [7, 109, 141]. The risk for developing increased fibrosis in NASH over 5 years is 25% and for developing cirrhosis is 15% [38, 97, 142]. The 5- and-10 year survival in NASH has been estimated at 67% and 59% respectively, although death may arise from comorbid conditions [38, 143]. NAFLD is a leading cause of “cryptogenic cirrhosis” in which aetiologically specific clinical, laboratory, or pathological features can no longer be identified [102, 144, 145]. NAFLD-associated cirrhosis can develop into subacute liver failure, progress to hepatocellular carcinoma, and recur posttransplantation [125, 144, 146]. Thus, it appears that NAFLD is associated with the entire spectrum of chronic liver disease: progressive fibrosis, cirrhosis, end-stage liver disease, and hepatocellular carcinoma. A number of risk factors have been identified as predictors for progressive fibrosis and cirrhosis in NAFLD including; BMI > 30, type II diabetes, age > 45 years, and an ALT : AST ratio > 1 [7]. 

Histologic improvement may also occur with weight loss, especially in those with only minimal fibrosis [147]. This is particularly true when weight loss is achieved slowly [147, 148]. Rapid weight loss has been noted to accelerate disease progression [101, 149, 150]. Liver failure becomes manifest in many cases during a period of rapid weight loss regardless of its mechanism [101, 149, 151].

It is clear that patients with NAFLD and even more so with NASH have a serious liver disease with a fibrogenic potential that can result in liver-related morbidity and mortality.

## 10. Visceral Fat and NAFLD

Despite the high prevalence of NAFLD and its potential for serious sequelae, the underlying aetiological factors that determine disease progression from simple steatosis to NASH and cirrhosis remain poorly understood. Disordered adipokine production is likely to play a role in the pathogenesis of NAFLD [106, 152]. Adipokines are implicated in the pathogenesis of NAFLD/NASH through their metabolic and pro-/anti-inflammatory activity [153]. The literature reveals quantitative and qualitative differences in the repertoires of mediators that are released from peripheral and visceral fat depots [154, 155] (Table 6). The net adipokine profile generated by visceral fat appears more noxious than that of subcutaneous fat [60]. Hence, visceral adiposity accompanies the metabolic syndrome [60, 154]. Increasing visceral obesity is thought to result in increased production of proinflammatory cytokines and adipokines [tumour necrosis factor alpha, interleukin 6, and C reactive protein] [26, 156–158] and decreased production of protective adipokines [adiponectin] [28, 153, 156]. This abnormal balance might ultimately lead to the clinical and histopathological occurrence of NASH. 

The exact role of adipokines in the pathogenesis of NAFLD, however, remains investigative. Literature reveals variable results about alteration in leptin [159–161], adiponectin, and TNF-*α* [153, 162] while the role of resistin in NAFLD in humans still awaits to be examined [162–164] though increased resistin levels have been correlated with NAFLD severity and NASH development [165, 166]. Investigators have shown a direct dose-dependent link between increasing amounts of visceral fat and end-organ tissue inflammation and fibrosis in patients with NAFLD demonstrating visceral fat to be directly associated with liver inflammation and scarring in the metabolic syndrome [166, 167]. Importantly, this effect was independent of levels of hepatic steatosis, patient age, and insulin resistance [166, 167]. Ethnic differences in visceral fat are being investigated as potential links to understanding differences in NAFLD. Compared to Caucasians, lower levels of hepatic triglycerides have been found in African Americans despite similar total body adiposity and insulin resistance [166, 168], whilst Asians have increased visceral fat depots in relation to their body mass index (BMI) risking NAFLD despite low BMI [166, 169].

## 11. Conclusion

The growing epidemic of obesity across the globe constitutes a major challenge to health services. Nonalcoholic fatty liver disease (NAFLD) has become the most common of all liver disorders with prevalence paralleling the degree of obesity. A clear understanding of the mechanisms underlying disease progression is urgently required to develop prevention and treatment strategies. Adipokines secreted by adipose tissue have recently been implicated in initiating and perpetuating the chronic inflammatory state observed in obesity and NAFLD. Studies should be undertaken to examine the exact role of adipokines derived from intra-abdominal fat as these appear to be the main drivers for the development of progressive liver injury in patients with severe obesity.

## Figures and Tables

**Table 1:** WHO classification of overweight and obesity [2].

Classification	Body mass index (kg/m^2^)	Associated health risks
Underweight	<18.5	Low (but risk of other clinical problems increased)
Normal range	18.5–24.9	Average
Overweight	25.0 or higher	
Pre-obese	25.0–29.9	Increased
Obese class I	30.0–34.9	Moderately increased
Obese class II	35.0–39.9	Severely increased
Obese class III	40 or higher	Very severely increased

**Table:** (a)

Central obesity	
Waist circumference*-ethnicity specific	

Plus any two of the following	

Raised triglycerides	≥1.7 mmol/L (150 mg/dL) Or speccfic treatment for this lipid abnormality

Reduced HDL-cholesterol	<1.03 mmol/L (40 mg/dL) in males <1.29 mmol/L (50 mg/dL) in females Or specific treatment for this lipid abnormality

Raised blood pressure	Systolic: ≥130 mmHg or diastolic: ≥85 mmHg Or treatment for previously diagnosed hypertension

Raised plasma glucose	Fasting plasma glucose ≥5.6 mmol/L (100 mg/dL) Or previously diagnosed type 2 diabetes If above 5.6 mmol or 100 mg/dL An oral glucose tolerance test is strongly recommended but is not necessary to define the presence of the syndrome

**Table:** (b)

Country/ethnic group		Waist circumference (cm) (as measure of central obesity)

Europeans	Male	≥94
	Female	≥80

South Asians	Male	≥90
	Female	≥80

Chinese	Male	≥90
	Female	≥80

Japanese	Male	≥85
	Female	≥90

**Table 3:** The Third US National Health and Nutrition Examination Survey (NHANES III) clinical criteria for the presence of metabolic syndrome (presence of ≥3 criteria) [25].

Criteria	Value
Abdominal obesity	Waist circumference >102 cm men and >88 cm women

Hypertriglyceridemia	≥150 mg/dL

Low high-density lipoprotein (HDL)	<40 mg/dL in men and <50 mg/dL in women

High blood pressure	≥130/85 mmHg

High fasting glucose	≥110 mg/dL

**Table 4:** Body fat distribution [27].

Depot	Remarks
Subcutaneous	About 80% of all body fat. Can functionally be divided into abdominal and gluteofemoral

Visceral	Drained by the portal vein. Anatomically divided into omental, mesenteric, and retroperitoneal fat

Other	Peritoneal and orbital

**Table 5:** Regional differences in lipolysis [27, 48].

Hormone	Action on lipolysis	Regional differences
Catecholamines	Stimulating	VF > SCF abd > SCF glf
Insulin	Inhibiting	SCF > VF
Prostaglandins	Inhibiting	SCF > VF
Adenosine	Inhibiting	SCF > VF

VF: visceral fat; SCF: subcutaneous fat; abd: abdominal; glf: gluteofemoral.

**Table 6:** Differences in adipocytokines expression between visceral and subcutaneous fat in humans [28, 38, 49–52, 57, 63–67, 75].

Adipocytokine	Differences in expression
Adiponectin	VF < SCF
Adipsin	VF < SCF
ASP	VF < SCF
CETP	VF < SCF
Leptin/Ob-Re	VF < SCF
TNF-*α*	VF = SCF
Angiotensinogen	VF > SCF
Factor B	VF > SCF
Il-6	VF > SCF
IL-8	VF > SCF
PAI-1	VF > SCF
PPAR-*γ*	VF > SCF
Resistin	VF > SCF
VEGF	VF > SCF
11*β*-HSD type 1	VF > SCF
MCP-1	VF > SCF
Visfatin	VF > SCF
Omentin	VF > SCF

11*β*HSD type 1: 11-hydroxy-steroid-dehydrogenase type 1; ASP: acylation stimulation protein; CETP: cholesterol ester transfer protein; IL-6: interleukin-6; IL-8: interleukin-8; PAI-1: plasminogen activator inhibitor-1; PPAR-*γ*: peroxisome proliferators-activated receptor-*γ*; SCF: subcutaneous fat; TNF-*α*: tumour necrosis factor-*α*; VF: visceral fat; VEGF, vascular endothelial growth factor; MCP-1: monocyte chemoattractant protein-1.

**Table 7:** Grading and Staging of NAFLD [79, 90, 92].

*Grading NAFLD*	
(1) Macrovesicular steatosis	
Grade 0: None	
Grade 1: Up to 33%	
Grade 2: 33%–66%	
Grade 3: >66%	

(2 ) Necroinflammatory activity	
Grade 1 (mild)	Steatosis up to 66%, occasional ballooned hepatocyte (mainly zone 3), scattered intra-acinar neutrophils ± lymphocytes, no or mild portal inflammation
Grade 2 (moderate)	Steatosis of any degree, obvious zone 3 ballooning degeneration, intra-acinar neutrophils, zone 3 perisinusoidal fibrosis may be present, mild to moderate, portal and intra-acinar inflammation
Grade 3 (severe)	Pan-acinar steatosis, widespread ballooning, intra-acinar inflammation, neutrophils associated with ballooned hepatocytes, mild to moderate portal inflammation

*Staging NAFLD*	

(1) Stage 1	Zone 3 perisinusoidal/pericellular fibrosis; focally or extensively present
(2) Stage 2	Zone 3 perisinusoidal/pericellular fibrosis with focal or extensive periportal fibrosis
(3) Stage 3	Zone 3 perisinusoidal/pericellular fibrosis and portal fibrosis with focal or extensive bridging fibrosis
(4) Stage 4	Cirrhosis
